# The speed-gene study: methods, study design and preliminary results

**DOI:** 10.1186/s13104-023-06617-3

**Published:** 2023-11-22

**Authors:** Swam Htet, Miftahul Zannah, Thet Hnin Moe, Pongpanot Wongveerakul, Nongnapas Charoenpanich, Vitoon Saengsirisuwan, Ioannis Papadimitriou

**Affiliations:** 1 Department of Physiology, Faculty of Science, Mahidol University, Bangkok, Thailand; 2 Human Movement Performance Enhancement Research Unit, Faculty of Sports Science, Chulalongkorn University, Bangkok, Thailand

**Keywords:** Torque, Power, Biomechanics, DNA, Physiology

## Abstract

The Speed-Gene study aims to identify genetic variants influencing athletic performance and human locomotion using motion capture technology. Currently, 60 female participants have completed the testing protocol, and the overall aim is to recruit 283 moderately trained, healthy Southeast Asian individuals (18–45 y, BMI < 30). Participants will undergo biomechanical analysis and genetic testing. Several analyses will be performed, including (but not limited to) linear and angular kinematic analysis using motion capture technology, force plate dynamometry and genetic analyses to define novel power/torque related outcomes that would be more sensitive to allele-specific differences in athletic performance. Pretesting beverages will be provided, and activity history and current activity levels will be assessed by a questionnaire. The kinematic data will be obtained using a Qualisys Track Manager (QTM) system, and DNA will be extracted from white blood cells. The participants serve as their own controls. Although the Speed-Gene study is tightly controlled, our preliminary findings still indicate considerable individual variability. More participants and further genetic analysis are required to allow the investigation of potential underlying genetic mechanisms responsible for this individual variability.

## Introduction

Over the last 30 years significant research has been conducted to uncover the genetic basis of athletic performance and health related fitness. Athletic performance is influenced by phenotypes, such as body size, explosive strength, muscle flexibility and maximal oxygen uptake, each of which results from a wide variety of genetic, physiological, anthropometric, and biomechanical factors, as well as their interactions [1]. It is believed that these traits are affected by the inheritance of over 20,000 genes, among which ACTN3 and ACE are two of the most studied genes in relation to athletic ability. Numerous studies employed primarily the candidate gene approach have demonstrated that individuals who lack α-actinin-3 protein in their muscles (ACTN3 XX genotype) or produce low amounts of angiotensin-converting enzyme (ACE II genotype) in their body are less successful in speed-related sports [2–4].

Furthermore, several association studies have demonstrated that α-actinin-3 protein and angiotensin-converting enzyme have beneficial effects on abilities related to speed and power, including short-distance sprint time and vertical jump height [5, 6]; however other studies do not show these effects [7, 8]. Among these studies, some were limited by sample heterogeneity and size. Others were constrained because the abilities of muscles to create explosive speed and power were not assessed using laboratory analyses, but rather using less accurate field tests [9]. Recent genome-wide association studies (GWAS) [10–12], identified only few gene variants that associate with physical performance and the genetic underpinnings of athletic prowess still remain largely elusive. This unexplained heritability could be partly due to targeting more complex athletic phenotypes such as athletic status or measuring less specific performance outcomes that involving multiple genes and their interactions.

In this study, we aim to overcome these limitations and provide new insights into the influence of genetics on performance and human physiology. To this end, we will apply motion capture technology to explore how various genetic polymorphisms may affect more specific biological characteristics during explosive SSC-type movements. The overall goal of this study is to identify gene variants that influence torque production on certain joints and impact on various specific power related outcomes during explosive body movements as shown in Table 1. crossing scientific specialties and using current state-of-the-art technology. This study design has the potential to provide novel torque/power related outcomes that would be more sensitive to allele-specific differences.

## Methods

Sprinters, power-oriented athletes, and anyone who regularly engage in heavy resistance training or sports related to sprinting will be excluded from our analysis. To ensure both body composition and genetic homogeneity, only female, Southeast Asian (for 3 generations) volunteers with a mean body mass index (BMI) of 23 kg/m^2^ would be recruited for this study.

Participants will be excluded from the study if they respond “yes” to any of the eight PAR-Q questions or have any conditions that restrict their ability to exercise, such as high blood pressure or musculoskeletal disorders. The GPPAQ questionnaire will also be used for evaluating our cohorts’ current levels of physical activity. Participants whose work demands intense physical activity, such as handling very heavy objects (such as workers in the construction industry, scaffolders, and refuse collectors), or participants who reported participating in power strength-related recreational activities, such as gym training, and tennis will be excluded.

Seventy-two hours prior and throughout all tests, participants will be asked to abstain from coffee, alcohol, drugs, caffeine or other stimulants. Participants will be required to consume an energy drink containing mostly liquids and carbs (1 to 1.5 g•kg1 BM) before to the start of the test in order to allow appropriate access to carbohydrate energy stores.

### Study overview

**Fig. 1:**
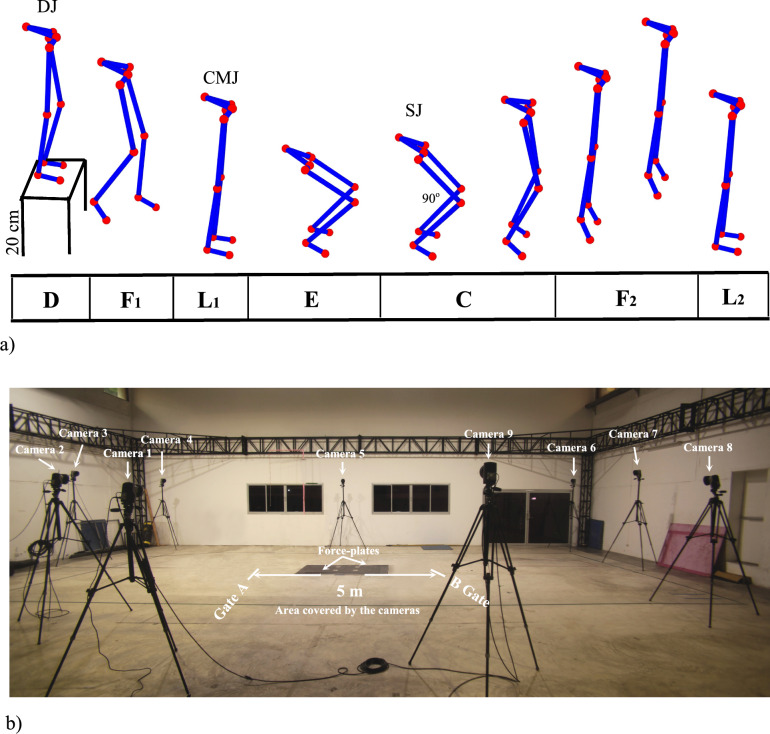
**a** Initial DJ position (D), flight time (F1), landing (L1), eccentric contraction (E), concentric contraction (C), flight time (F2), and landing (L2). CMJ initial position (L1), E: Eccentric contraction (C), flight time (F2), landing (L2), SJ initial 90^o^ position (C), concentric contraction (C), flight time (F2), landing (L2).** b** The locations of nine cameras, two marker gates (Gate A & Gate B), and the force-plates

**Fig. 2:**
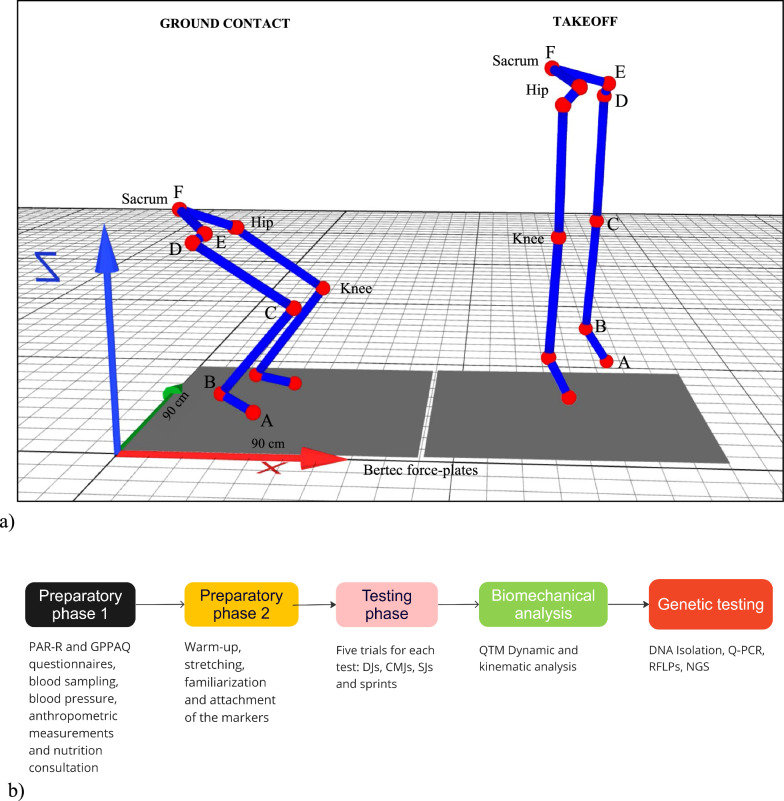
**a** An overview of the eleven skin-marker model that will be implemented using a snapshot of the QTM program. Point (A) indicates the fifth metatarsal of the distal foot. Point (B) illustrates the apex of the lateral malleolus of the fibula. Point (C) displays the lateral condyle of the femur. Point (D) indicates the femoral greater trochanter. Point (E) displays the anterior superior iliac spine. Point (F) demonstrates the sacrum. The force plates will provide dynamic data from take-off, which will enable QTM software to calculate the maximal velocity and acceleration in the hip (EDC) and knee (BCE) joints.** b** Study Overview. Each participant will be asked to complete the PAR-Q and GPPAQ questionnaires, and 50–150 µl of blood will be drawn from their fingertips. Then, participants’ blood pressure, height, and weight will be measured for BMI calculation. A warm-up session consisting of stretching will be subsequently conducted, followed by the familiarization phase

Each participant will receive test instructions one week prior to testing. As previously described [13] two hours prior to testing, participants will be asked to practice squat jumps (SJ), counter movement jumps (CMJ) and, drop jumps (DJ), as illustrated in Fig. 1a, and an explosive 5-m sprint, as shown in Fig. 1b. After this practice time to familiarize themselves with the tests, the participants will perform five trials of each test. Figure 2b illustrates the study timeline.

As illustrated in Fig. 1a, SJ will be performed with parallel 90° foot alignment (Phase C) measured with a portable goniometer, followed by a restricted arm motion jump (Phase F2) in accordance with the method outlined in [9, 13, 14]. To perform CMJ, participants will stand upright (Phase L1), make a quick preparatory downwards movement, and then perform knee and hip flexion (Phase E) and extension (Phase C) to vertically propel their body from the ground (Phase F2). On the other hand, the DJ will be performed by standing on a 20 cm box (Phase D), stepping off (Phase F1), hitting the ground, and immediately jumping up as high as possible with a very short ground contact time (Phases E, C and F2) using an accelerated muscular contraction velocity combining rapid coupling between an eccentric and concentric muscle action, commonly known as a stretch-shortening cycle (SSC). 5-m sprint tests will be performed between gates A and B, as illustrated in Fig. 1b.

Data will be collected using motion capture technology, and processed using Qualisys Track Manager (QTM). Eleven reflective markers will be placed at precise anatomical sites on the trunk and lower extremities. In particular, they will be positioned on the sacrum (F), anterior superior iliac spine (left and right) (E), greater trochanter (left and right) (E), lateral condyle (left and right) (C), apex of the lateral malleolus (left and right) (B), and fifth metatarsal (left and right). Each of the nine cameras will have its exposure, threshold, frequency, and flash duration adjusted to precisely detect the reflecting marker position. If a marker’s reflection is unsatisfactory, we will adjust its threshold, exposure, or flash time to ensure that we can effectively observe the marker’s reflection on the computer screen. The wand and L-shaped structure supplied by the manufacturer will be used to calibrate the equipment prior to each testing session.

The captured data from the reflecting marker (F) will be utilized to analyse kinematic data such as jump height, maximum velocity, acceleration and running times. The recorded data from reflective markers (C, D, and E) will be used to identify the angular velocity and acceleration of the hip joint, while the data collected from reflective markers (B, C, and D) will be used to detect the angular velocity and acceleration of the knee joint. For the measurement of torque in hip or knee joints, segmental mass will be multiplied by angular acceleration [15], following the method previously described [16] according to the following formulas shown in Table 1.

On the basis of the model shown in Fig. 2a, eleven reflective markers will be attached to precise anatomical locations with adhesive tape as previously described [13]. Briefly, during the familiarization phase, the participants will perform five trials for each type of jump (squat, counter movement, and drop jumps), as well as the 5-m sprint, followed by 30–45 min of recovery. After this recovering time, the testing phase will begin. Using motion capture technology, the subjects’ movements will be captured during their performance of five trials for each jump and the 5-m sprint. All participants will be allowed three minutes of rest between each session. After testing, the data obtained from dynamometers and cameras will be processed using QTM software.

As shown in Fig. 2a, we will utilize a Bertec force plate (90 cm x 90 cm) to measure force-related dynamic parameters. The following table demonstrates the outcomes of interest that will be measured and indicates the formulas that will be used to calculate each parameter.

**Table 1:** The parameters of interest and their calculation formulas are summarized in the table

	Variables	Formulas	Outcomes	References
**Angular**	**Relative Torque**	$RT = \displaystyle{\frac{{Segemental\,mass\,(kg)\, \times \,Angular\,accelerations\,(\deg \,{s^{ - 2}})}}{{Total\,body\,mass\,(kg)\,}}}$	Torque and power production	Knudson [15]
	**Power**	$P\, = \,\operatorname{Re} lative\,Torque\,(Nm)\, \times \,Angular\,velocity\,(\deg \,{s^{ - 1}})$		Sayers et al., [17]
**Dynamic**	**Peak Power**	$P\, = \,\operatorname{Re} lative\,Torque\,(Nm)\, \times \,Angular\,velocity\,(\deg \,{s^{ - 1}})$	Explosiveness, and force production	Turner et al., [18]
	**Rate of Force Development**	$\displaystyle{RFD\, = \,\frac{{Peak\,Force\,(N)}}{{Time\,to\,peak\,Force\,(s)}}}$		Comfort et al., [19]
**Reactive**	**Reactive Strength Index**	$\displaystyle{RSI\, = \,\frac{{DJ\,Height\,(m)}}{{Ground\,Count\,Time\,(s)}}}$	Eccentric force utilization during SSC	Riggs & Sheppard, [20]
	**Index of Reactive Force**	$\displaystyle{I\operatorname{Re} aF\, = \,\frac{{DJ\,Height\,(m)\, - \,SJ\,Height\,(m)}}{{SJ\,Height\,(m)}}}$		Papadopoulos et al., [14]

The angular variables are quantifying hip and knee angular acceleration and velocity during jumps and sprints while the dynamic variables quantifying various force-time curve characteristics. The reactive variables demonstrate the elastic utilization when eccentric muscle contractions are followed immediately by explosive concentric contractions in vertical jumps during SSC

### Genetic analysis

A spring-loaded lancet will be used to pierce the participant’s index finger tip skin in order to collect a small amount of blood (80–150 µl) as previously described [21]. The DNA extraction will take place at room temperature, and the isolated DNA (yield: 10–20 µg) will be stored at -18 °C. Polymerase chain reaction (PCR) will be used for DNA amplification. A number of genetic tests will be carried out, including quantitative Q-PCR, restriction fragment length polymorphism (RFLP), and next generation sequencing (NGS).

### Sample size and statistical analysis

The calculation of sample size is based on a conservative estimate of both the expected variation and the standard deviation for torque and power related measurements derived from our pilot work [13]. Our power analysis was conducted with G-Power software and indicates a total sample size necessary to achieve statistical significance using an alpha of 0.05, effect size set at 0.25 and a power of at least 0.80 is 269 participants. A cohort of this size will allow us to override the confounding effects of genetic and environmental heterogeneity including main effects and interactions. Assuming that 5% do not complete the experiment (e.g., drop out or are injured), the total sample size will be increased to a total 283 participants. We note that this sample size is 2.0 times that of the kinematic study on which we based our power analysis [22]. To test our hypothesis we will recruit a prospective human cohort with 283 participants in which age, training state, nutrition, BMI, ethnic background and gender will be tightly controlled.

To compare the effect of each performance parameter initially we will use the one-way analysis of variance (ANOVA). The Tukey’s post-hoc test will be used to determine statistical significant difference among the genotype groups. The level of significance will be set at 0.05. Then the quantitative genetic association data and performance parameters will be analysed using linear regression models, with related torque and power outcomes and alleles as covariates. As previously described [6] using the Simple Interactive Statistical Analysis website (SISA; www.quantitativeskills.com/sisa/) the genotype interactions on performance parameters will be further assessed using correlation analysis. Briefly, three genetic models (additive model and two dominant models assuming complete dominance of each allele) will be tested. The additive genetic model will be consisted of 0, 0.5 and 1, to represent I allele homozygotes, ID heterozygotes and homozygotes for the D allele, respectively; for the I allele dominant or D allele dominant genetic models, the corresponding values will be 0, 0, 1 or 0, 1, 1, respectively. The percentage of the genetic contribution to phenotypic variance explained by each genetic model will be estimated by expressing r^2^ from the correlation analyses (taken as an estimate of percentage variance explained under the model) as a percentage of the variance explained by genotype effects in the model-free ANOVAs, as previously described [22]. This proportion will be compared for each model to predict the most accurate model tested. Furthermore, to test the effect of each performance parameter shown in Table 1a full model containing all covariates for each type of variable will be compared with a null model missing the covariate of interest, using a likelihood ratio test. Males and females will be analysed separately.

For the preliminary results, a t-test was utilized for the comparison of the mean values between the female participants with different ACTN3 and ACE genotypes. To check the normality, we used the Kolmogorov Simonov test. The observed genotype counts were not significantly different from those expected under Hardy–Weinberg equilibrium (HWE).

### Results

**Fig. 3:**
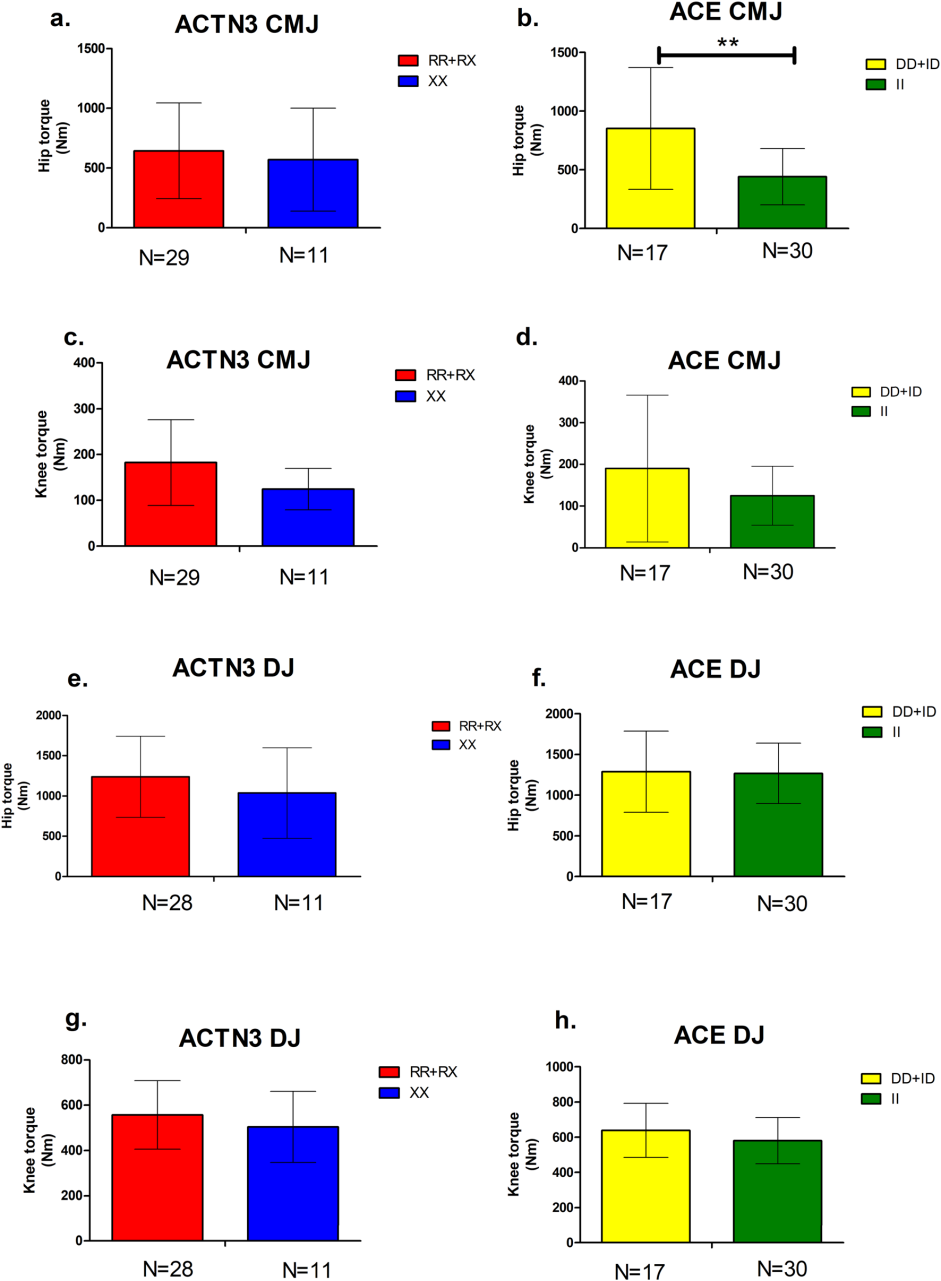
**a** Relative hip torque production in the CMJ eccentric phase, compared between groups of female participants based on ACTN3: *RR + RX* vs. *XX*.** b** Relative hip torque production during the CMJ eccentric phase, compared between groups of female participants based on ACE: *DD + ID* vs. *II*.** c** Relative knee torque production in the CMJ eccentric phase, compared between groups of female participants based on ACTN3: *RR + RX* vs. *XX*.** d** Relative knee torque production during the CMJ eccentric phase, compared between groups of female participants based on ACE: *DD + ID* vs. *II*.** e** Relative hip torque production during the eccentric DJ phase, compared between groups of female participants based on ACTN3: *RR + RX* vs. *XX*.** f** Relative hip torque production during the DJ eccentric phase, compared between groups of female participants based on ACE: *DD + ID* vs. *II*. g) Relative knee torque production during the DJ eccentric phase, compared between groups of female participants based on ACTN3: *RR + RX* vs. *XX*. h) Relative knee torque production during the DJ eccentric phase, compared between groups of female participants based on ACE: *DD + ID* vs. *II*.

The preliminary results shown in Fig. 3 demonstrate that, female participants with the ACE DD + ID genotype exhibit increased torque production of knee during the CMJ eccentric phase, compared to their ACE II counterparts as illustrated in Fig. 3b. Furthermore, ACTN3 RR + RX female participants demonstrated a trend towards larger amounts of hip torque during the eccentric phase of DJs compared to their ACTN3 XX counterparts, as indicated in Fig. 3e.

## Conclusions

Considering the complexity of athletic performance it seems evident that it does not fit straightforwardly into a single disciplinary framework and cross-disciplinary research is necessary. The planning and development this study combining biomechanical and genetic analysis began in 2020. Subsequently, recruitment and data collection were initiated at Mahidol University. To date, 60 female participants have completed the testing protocol, and 100 have completed the DNA isolation analysis. Although our study was tightly controlled, we still observed significant variability.

The preliminary results demonstrate that, compared to their ACTN3 XX counterparts, ACTN3 RR homozygotes demonstrated a trend towards higher hip torque production within the DJ eccentric phase. Furthermore, female participants with the ACE DD genotype exhibit increased knee torque production within the CMJ eccentric phase compared to their ACE II counterparts, indicating the need for further research in this area.

Recent in vivo evidence indicates that the ACTN3 gene influences the preservation of force with increasing muscle contraction velocity, rather than force itself [23]. Based on ground contact time, CMJs have been described as slow (> 250 milliseconds), while DJs are described as fast (< 250 milliseconds) [9]. A DJ performed by standing on a box, stepping off, hitting the ground, and immediately jumping up as high as possible with a very short ground contact time, using an increased muscle contraction velocity combined with explosive coupling between an eccentric and concentric muscle action, commonly known as a stretch-shortening cycle (SSC). The presently available data lead us to hypothesize that the ACTN3 R577X genotype could have a more pronounced effect on jumps performed at a higher muscle contraction velocity (such as DJs) whereas ACE gene possibly on slower velocity jumps (such as CMJs). Furthermore, data from our male cohort show evidence that participants with the ACTN3 RR genotype exhibit increased torque production during DJs compared to their ACTN3 XX counterparts [13], indicating the need for further research.

These results align with recent research findings obtained using isokinetic dynamometry tests that demonstrated that ACTN3 RR individuals exhibit larger peak torque at higher angular speeds (30–180 deg/s) than ACTN3 XX individuals [24]. Isokinetic dynamometers are devices that resist applied forces and control the angular velocity of a specific joint at a predetermined rate. We propose that angular kinematic analysis during multijoint type of movements has many advantages over the analyses used in previous studies and it could be a more sensitive way to detect small allele effects on muscle power performance characteristics. Firstly, emphasises on individual joints without the direct influence of the total anthropometric characteristics of the human body influencing the measurement. Secondly, more specific data such as peak torque and muscle power on specific joints can be precisely analysed during these explosive types of body movements. Therefore, assessments using isokinetic dynamometry are limited to single-joint tasks with a fixed angle of movement. However, our methodological approach using motion analysis technology allows the assessment of more specific muscular characteristics and provides a novel study design in the field of Sport Genomics.

Since DJs are considered a functional indicator of the explosive muscle power of leg extensor muscles, the difference detected in the hip joint enables us to hypothesize that the α-actinin-3 protein is likely to affect the extensor muscle group kinematic chain during the eccentric phase of these jumps. Within the DJ eccentric phase, noncontractile muscle supports contractile muscle, thereby increasing muscle force [25]. Accordingly, we propose that during the eccentric phase, the α-actinin-3 protein might influence how much elastic energy can be stored by type II sarcomeres. On the other hand the observed increased torque production of *ACE DD + ID* female participants during CMJs compared to their *ACE II* counterparts demonstrates the polygenic nature of peak athletic performance, with each gene influencing certain performance characteristics. When there is large number of genes involved, it becomes hard to distinguish the exact effect of each individual gene variant on physical performance. Previous studies [6, 22]- [24] have not analysed specific variables considered key factors for achieving high levels of power performance, such as maximal angular velocity, torque production, and acceleration in SSC during multijoint type of movements. Our approach, adapting motion capture technology for use in the fields of exercise physiology and muscle genomics, crossing the scientific specialities, aim to investigate the influence of certain gene variants on torque production and other specific force-time curve characteristics among Southeast Asian individuals. More participants are required to further investigate this hypothesis and provide sufficient statistical power to apply a more advanced statistical and genetic analysis. With further research, the designed study can provide evidence that specific genetic polymorphisms influence the body’s ability to produce greater amounts of power and torque during explosive and powerful contractions.

More participants are being recruited to provide sufficient statistical power to investigate both specific (hypothesis-driven) and nonspecific (hypothesis-free) genetic variants as possible predictors, allow follow-up of these results with appropriate statistical analyses, and to expand the genetic analysis to include additional genetic loci and techniques.

## Data Availability

The data that support the preliminary findings of this study are available on request from the corresponding author.
